# Reef fish escape responses selectively match predator attack speeds

**DOI:** 10.1371/journal.pone.0346465

**Published:** 2026-08-12

**Authors:** Sara Linde Neven, Lena Faber, Benjamin T. Martin

**Affiliations:** 1 Department of Theoretical & Computational Ecology, University of Amsterdam, Amsterdam, The Netherlands; 2 Department of Ecology and Evolutionary Biology, University of Colorado, Boulder, Colorado, United States of America; University of Ferrara, ITALY

## Abstract

Animals must continually balance foraging with the risk of predation. In complex natural environments, this means quickly distinguishing between threats and harmless situations. We investigated how site-associated coral reef fishes escape in response to visual cues mimicking predator attacks, using controlled underwater presentations of looming stimuli at varying speeds. We measured escape responses across species and social contexts, and compared them to predator attack speeds observed in the same habitat. Escape probability increased sharply with the speed of the looming stimulus, with no responses at low speeds. Stimulus speeds that triggered escape responses matched those of predator attacks, whereas speeds similar to those of cruising predators never triggered a response. The two species differed in behavior: Brown Chromis ranged farther from shelter and responded more readily, whereas Bicolor Damselfish stayed close to shelter and responded less. Contrary to expectations, social context did not affect responses. These findings demonstrate that reef fish are highly sensitive to the approach speed of objects, with species-specific behavior further shaping their responses. By combining realistic visual threats with natural predator attack data, this study offers insight into how animals make rapid escape decisions in complex, real-world environments.

## Introduction

Wild animals regularly face threats from predators, and rapid threat detection and response are crucial for survival [1]. Prey must quickly assess their surroundings and initiate escape when necessary in order to reach safety in time [2]. While these assessments are challenging, prey are remarkably successful at them, a phenomenon reflected in the low capture success rates of predators reported in aquatic systems [3–5].

However, escape decisions come with a trade-off. Unnecessary responses waste energy and interrupt foraging opportunities [6]. Failing to respond can be fatal, but fleeing too often is energetically costly. Successful decision-making therefore depends on reliably distinguishing threatening from non-threatening situations, and on adjusting behavior based on information about the surroundings [2].

One ecosystem where these decisions are especially common is the coral reef, where predators and prey encounter one another frequently [7]. These environments pose a challenge for accurate, rapid escape decisions. Prey must pick out approaching predators against a crowded moving backdrop of conspecifics and other non-threatening species. Moreover, the predators inhabiting coral reefs employ diverse strategies to intercept their prey, including pursuit, ambush, and stalking [8,9]. While these tactics differ in the precise mechanics of capture, they share a key feature: predators generally approach rapidly in the terminal phase of the attack. To survive, prey must detect and quickly respond to these attacks while suppressing escape responses to non-threatening stimuli.

Fish detect approaching predators through several channels, including rapid pressure changes [10], sound [11], and vision. Among these, visual cues are especially important for triggering escape in many species [12,13]. A looming stimulus, the expanding retinal image of an approaching object, reliably evokes escape across a wide range of taxa, from fish to insects, birds, and humans [14,15]. In fish, fast-approaching objects excite the Mauthner cell pathway, which triggers a rapid, stereotyped escape known as a C-start [16–19]. This pathway is tuned to approach speed: fast approaches elicit the reflexive C-start, whereas slower approaches are less likely to activate it, allowing the fish to integrate additional information, such as the presence of a refuge or of neighbors, before responding [20–23].

Whether fish in the wild actually use approach speed to tell a genuine threat from harmless movement, however, remains unclear. Most of the evidence that escape depends on speed comes from the laboratory [16,17]; it is untested whether, in their natural environment, fish flee from the fast approach of a real attack while ignoring the slower movements of a cruising predator. On coral reefs, predators are often within sight of their prey but rarely attack [7], so prey must repeatedly assess whether an approach is dangerous. Because an attack is several times faster than routine cruising [24], the speed of an approaching predator offers a reliable cue for making that judgment. We therefore expect escape to become more likely as stimulus speed increases, and we test whether the speeds that trigger escape match those of real attacks rather than routine cruising.

Escape decisions also depend on ecological context. Many coral reef fish show strong site fidelity and rely on coral structures for shelter [25,26]. Because individuals farther from shelter take longer to reach safety, they are likely to flee more readily from an approaching threat. The two focal species of this study also differ in ways likely to shape these decisions. Bicolor Damselfish (*Stegastes partitus*) are territorial and strongly shelter-associated, defending small coral patches [27], whereas Brown Chromis (*Chromis multilineata*) are more mobile, foraging on plankton in the water column and ranging farther from shelter [25,26]. Because a mobile, exposed forager has more to lose by failing to flee, we anticipate a stronger escape response in Brown Chromis than in the shelter-bound Bicolor Damselfish.

Social context can further modulate escape. When prey occur in groups, the per-capita risk of capture is reduced through dilution, which can make any given individual less likely to flee in response to a threat [28–30]. We quantify social context as neighbor proximity, which increases with the number and closeness of nearby fish and reflects roughly how much of a focal fish’s visual field they occupy. Under the dilution hypothesis, this predicts that escape probability falls as neighbor proximity increases. In fishes, however, the evidence is mixed: some studies find that being in a group reduces individual responsiveness [29], whereas a recent analysis found only weak support for an effect of group size on antipredator responses [31].

Here, we pair controlled looming stimuli with observations of natural predator attacks to test how site-associated coral reef fish decide to escape in a complex, natural environment. By presenting looming stimuli across a range of speeds and comparing which speeds trigger escape against the attack speeds of a natural predator in the same habitat, we test whether prey selectively respond to speeds characteristic of attacks while ignoring those typical of routine swimming. We further ask how distance to shelter, species identity, and social context shape these responses, and whether responsiveness declines over repeated presentations through habituation. We also account for aspects of the experimental setup, a fish’s distance from and viewing angle to the screen, and its body orientation, that could affect its responses independently of the ecological factors of interest. By combining a field experiment with the natural distribution of predator attack speeds, this study reveals how prey integrate threat cues with ecological and social context to make rapid escape decisions.

## Materials and methods

### Data collection and video processing

#### Experimental setup.

**Fig 1:**
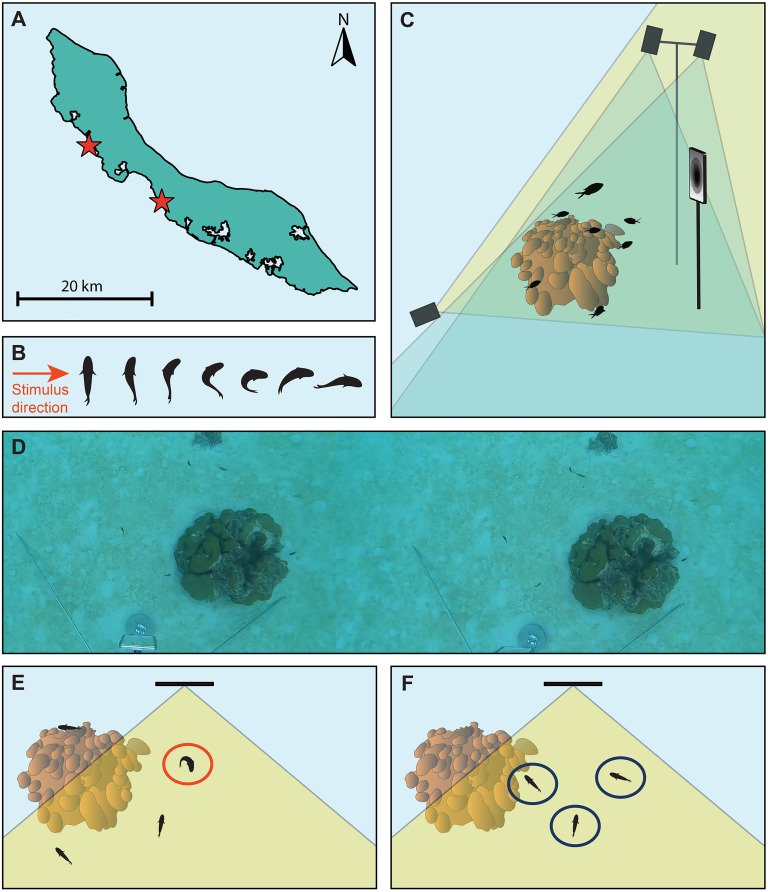
Overview of experimental setup. A: Locations of setup in Curaçao. Red stars mark the experimental sites at Cas Abou and Kokomo Beach. The island outline was derived from geoBoundaries data (https://www.geoboundaries.org), used under a CC BY 4.0 license. B: Top view of body postures during escape response (referred to as a C-start [18]). C: Experimental setup around an isolated coral patch, with two top-view cameras (blue line of sight) and one side-view camera (yellow line of sight). D: Top view from the left and right top-view cameras. E: First-responding fish (FR, red circle) in a response video. F: Non-responding fish that saw the stimulus (NRL-NR, blue circles) in a non-response video. Panels E and F show the two groups compared in the analysis.

The experiments were conducted on shallow reef flats at Cas Abou and Kokomo Beach along the west coast of Curaçao, in the Caribbean Sea (Fig 1A). A total of 12 trial sessions were conducted across 5 sites around the reef (all located at approximately 3 meters in depth), with each site sampled on one to four separate occasions. Each trial session took place on an isolated coral patch that serves as a refuge for various fish species. Since most fish remained close to the coral, we could reliably track individuals during each stimulus presentation. No natural predator attacks occurred during any of the stimulus presentations that we analyzed.

The two dominant species are Bicolor Damselfish, which are territorial and site-attached to small coral patches [27], and Brown Chromis, which form site-attached aggregations and forage on plankton in the water column above the reef [32]. In addition to these species, the coral patches are frequently visited by other reef-dwelling species that belong to various families of coral reef fish.

At the experimental sites, we set up an iPad (iPad Pro 2022, 12.9 inches) to display a looming stimulus every 90 seconds over an approximately one-hour trial session. Three cameras (GoPro HERO9, 1080p, 240fps) were positioned around the coral patch: two facing downward and one arranged for a side view facing the stimulus (Fig 1C). The looming stimulus consisted of an expanding black disk on a white background, simulating an approaching object (a predator) in the region in front of the iPad screen. The expansion rate of the disk was calibrated to match the optical expansion produced by a predator approaching at one of nine different speeds (0.5, 1.0, 2.0, 3.0, 4.0, 5.0, 6.0, 7.0, or 8.0 $\;m\;s^{-1}$), and the order of the loom stimulus speeds was randomized across presentations within each trial session. For a more detailed description of the looming stimulus, see S1 Appendix (Looming stimulus).

#### Ethics statement.

All fieldwork was conducted under the Curaçaoan Government’s Permit #2022/21467 to CARMABI. The reef sites are publicly accessible. Because no animals were handled, this research was determined not to require a license from the Head Animal Welfare Body of the University of Amsterdam, as set out in the Dutch Experiments on Animals Act.

#### Video analysis and 3D reconstruction.

The video data from the three cameras were synchronized, and all recordings were manually screened for the presence of escape responses. An escape response was defined by a body bend (a C-start; Fig. 1B) followed by a rapid increase in speed [18]. For each fish exhibiting an escape response, the frame corresponding to the C-start was recorded (Fig. 1D).

To reconstruct the 3D positions of the fish and their environment, 2D positions from the two downward-facing cameras were manually annotated using the open-source platform CVAT [33]. These 2D positions were triangulated into 3D coordinates based on stereo correspondence between paired cameras using calculated stereo parameters [34]. To obtain these parameters, the cameras were calibrated before each trial session by presenting a calibration checkerboard at various orientations and positions within the cameras’ field of view. Stereo calibration parameters were estimated using the Stereo Camera Calibrator app in MATLAB [35]. For a detailed description of the environmental reconstruction, see S2 Appendix (Coral reconstruction).

Each fish was assigned a unique ID, and its species and response frame (if applicable) were recorded. Fish-specific variables, such as distance to coral, were calculated using the reconstructed 3D positions of the fish and the environment. Some fish were excluded from analysis because they were not visible in one of the stereo cameras or left the field of view, which prevented the calculation of their 3D positions or speeds. Each looming-stimulus presentation was recorded as a single video across the three cameras. Videos were excluded when more than 33% of the tracked fish were unusable. After filtering, the dataset comprised 247 videos, 67 with at least one responding fish and 180 with no observed responses.

#### Measuring natural attack speeds.

To quantify the attack speeds of a natural predator of the two focal prey species, we conducted a separate recording effort, independent of the looming-stimulus experiment. Stereo cameras (GoPro HERO9, 1080p, 120fps) were deployed on the same reef, but as a distinct setup and at different times from the stimulus trials. From this dataset, 61 attacks by Bar Jacks (*Caranx ruber*) were isolated and analyzed. Each attack was tracked from the moment the Bar Jack entered the field of view or initiated an attack until the prey, typically a Brown Chromis, either evaded capture or was caught.

3D tracks of the Bar Jack and Chromis were manually annotated by tracking head positions. Attack speed data were computed by calculating the magnitude of the change in position per frame and dividing it by the frame rate. The 90th percentile of the predator’s instantaneous speed was used as a proxy for maximum attack speed. This measure captures the fast-approach phase of the attack, which is most relevant to prey escape decisions, while reducing sensitivity to tracking noise associated with using absolute maxima.

### Calculation of variables

To analyze which factors shaped escape decisions, we calculated a set of explanatory variables (see S1 Table for an overview). These variables describe both experimental manipulations and natural variation in the context of the fish.

Our primary predictor was the programmed speed of the looming stimulus (LSp), the speed at which the simulated predator approached. In principle, one could instead characterize the stimulus by the speed or size of its image as perceived by the fish. However, because the looming image expands continuously, these perceived quantities take a value only at a particular instant. For a fish that responded, that instant is well defined (the frame of the C-start, accounting for the sensory-motor delay), but for a non-responding fish there is no comparable reference point. We therefore used the programmed speed, which is defined the same way for every fish, whether or not it responded.

The order of stimulus presentations within a trial (trial event number; TEv) was included to test for habituation, defined as a reduction in responsiveness to repeated stimuli. While habituation (a decrease in response to repeated stimuli) is commonly observed in laboratory animals [36], it tends to be slower and more context-dependent in wild animals [37].

Several variables captured how well each fish could perceive the stimulus. The distance to the stimulus (DSt) was measured as the three-dimensional distance between the fish’s head and the center of the iPad screen. Because the apparent size of an object scales with the inverse square of distance, fish farther from the iPad experienced a much smaller visual stimulus than those nearby [38]. The viewing angle described the angle at which the fish viewed the iPad screen. A viewing angle of $0^{\circ }$ corresponds to a fish directly in front of the iPad screen, with the angle increasing as the fish moves more to the side. At larger angles, the screen appeared dimmer, and it became reflective at a large enough angle ($\begin{array}{c} ≳50^{\circ } \end{array}$) due to a Snell’s window effect [39]. The orientation angle (OA) describes the fish’s body alignment relative to the stimulus, ranging from $0^{\circ }$ when facing the iPad directly to $180^{\circ }$ when facing directly away. This variable was included to account for the possible effect of predator approaching angle on escape probabilities.

Species identity (Sp) was manually identified from the video footage and included to assess interspecific differences in escape behavior. Distance to coral (DCo) was measured as the shortest three-dimensional distance to the nearest coral patch, which serves as a potential refuge. Neighbor Proximity (NPro) was calculated as the sum, over all neighboring fish, of the inverse squared distance to each neighbor. Because it is a sum, NPro increases both with the number of neighbors and with their closeness, with nearby individuals contributing more than distant ones. For fish of similar size, NPro is approximately proportional to the total area that neighbors project onto the focal fish’s retina, since a neighbor’s retinal image scales with the inverse square of distance [13,38,40].

### Dataset: classification of individuals

We analyze only responses to the looming stimulus itself; fish that reacted to their neighbors instead are not examined here. To isolate stimulus-driven responses, we classified fish by the information available to them, comparing first responders in the response videos (FR, Fig 1E) with fish that saw the stimulus but did not respond, in the non-response videos (NRL-NR, Fig 1F). Both groups comprise fish whose neighbors did not respond, so their behavior could not have been driven by the responses of others. A full description is given in S3 Appendix (Classification of individuals).

In response videos, first responders (FR, n = 95) were the fish that initiated the escape in each video, together with any fish responding within five frames (20.83 ms) of them. This window is the sensory-motor delay required to process a visual stimulus [41], and matches the shortest interval we observed between a first responder and a secondary responder that could not itself see the screen. Responses within it are therefore too fast to be triggered by a neighbor and instead reflect direct responses to the stimulus. This is a conservative criterion: it may exclude some genuine but slower stimulus-driven responses, but ensures that included responses are not socially driven. Fish responding more than five frames after the first responder were classified as secondary responders and excluded, because their responses could not be attributed unambiguously to the stimulus.

Classification also depended on whether a fish could see the screen. Beyond a viewing angle of roughly $55^{\circ }$, the iPad becomes reflective through the Snell’s window effect [39] and the stimulus is no longer visible. Among excluded secondary responders, those outside this $55^{\circ }$ window could only have responded to social information from neighbors, whereas those within it may have responded to the stimulus, their neighbors, or both (S3 Appendix). For non-response videos, we included only fish within $55^{\circ }$ of the screen, which therefore had a clear view of the stimulus, classifying them as NRL-NR (*n* = 1255).

### Statistical analysis

We modeled whether an individual fish initiated an escape response, a binary outcome, using generalized linear mixed models (GLMMs) with a binomial error distribution and a logit link, fitted in R (version 4.5.1) with the lme4 package [42]. Data processing was performed in Python (version 3.13.6). All models included a random intercept for trial session ID, because the data were unbalanced and hierarchically structured, with unequal numbers of observations across the 12 sessions and five sites (S1 Fig); this random effect strongly improved fit and was retained throughout (S5 Appendix). Continuous predictors were scaled to a mean of zero and a standard deviation of one to standardize effect sizes and improve convergence.

The fixed effects were the eight predictors defined above (S1 Table): stimulus speed (LSp), distance to the stimulus (DSt), viewing angle (VA), orientation angle (OA), species (Sp), distance to coral (DCo), neighbor proximity (NPro), and trial event number (TEv). Collinearity among the continuous predictors was low (all pairwise |*r*| < 0.35; S4 Appendix, S8 Table), so all predictors were retained. The relationship between species identity and distance to coral is examined in the Results.

To characterize each effect, we fitted single-predictor models and then a full multivariable model including all predictors simultaneously, interpreting each predictor’s partial effect. We further tested whether continuous predictors were better described by non-linear functional forms, examined a set of pre-specified two-way interactions, and assessed the contribution of individual predictors to the species effect. Across these steps we evaluated eight single-predictor models (S2 Table), the multivariable model (S3 Table; refit with two alternative measures of social context, S7 Appendix), four functional forms for each of the seven continuous predictors (S4 Table), six two-way interactions (S7 Table), and eight species-effect models (S6 Table).

#### Non-linear effects of single variables.

To account for potential threshold effects or other non-linear relationships between predictors and response probability, each continuous variable was tested in multiple functional forms by replacing it in the multivariable model with alternative transformations. Each predictor was tested in four forms: linear, logarithmic, and natural splines with two and three degrees of freedom, while all other predictors were initially held in their linear form. Model fits for these alternatives were compared using the Bayesian Information Criterion (BIC) to identify the best-fitting transformation for each variable. To evaluate the influence of non-linear transformations in the context of other non-linear variables, each variable was tested in a multivariable model where the other variables were in their optimal functional form.

#### Interaction effects.

To identify potential interactions between predictors, a list of ecologically relevant interactions was systematically tested using a multivariable model that incorporated the previously identified non-linear transformations of individual predictors. Each interaction term was added individually to the base model and compared to it using a likelihood ratio test (LRT) to evaluate whether the inclusion of the interaction term significantly improved the model fit. To account for multiple testing and control the false discovery rate, the raw p-values were adjusted using the Benjamini-Hochberg procedure [43].

To limit the number of interaction terms and focus on biologically meaningful combinations, we considered only two-way interactions among four ecologically relevant variables: looming stimulus speed (LSp), distance to the coral refuge (DCo), species identity (Sp), and neighbor proximity of neighboring fish (NPro). These variables were chosen based on a priori expectations that their effects on escape responses might be modulated by one another, reflecting trade-offs between environmental risk, social context, and species-specific behavior.

#### Species differences.

To evaluate whether species differences in escape probability could be attributed to other predictors, the effect of additional predictors on the species effect was tested. The species-only univariable model was compared to the same model extended with each individual predictor. For each model, the Akaike Information Criterion (AIC) and species coefficient estimates were compared. A reduction in the species coefficient, or a loss of significance, was interpreted as evidence that the added predictor accounted for part of the species effect. AIC was used here rather than BIC, as the aim was to track changes in the species coefficient when predictors were added rather than to select between competing model structures.

## Results & Discussion

Our results show that escape responses were dominated by the speed of the stimulus, and further influenced by spatial positioning and species identity. Contrary to expectations, social context had no measurable effect. Together, these results suggest that escape decisions in reef fish are governed primarily by information about the threat itself.

### Threat characteristics dominate escape decisions

#### Stimulus speed is the primary driver of escape responses.

**Fig 2:**
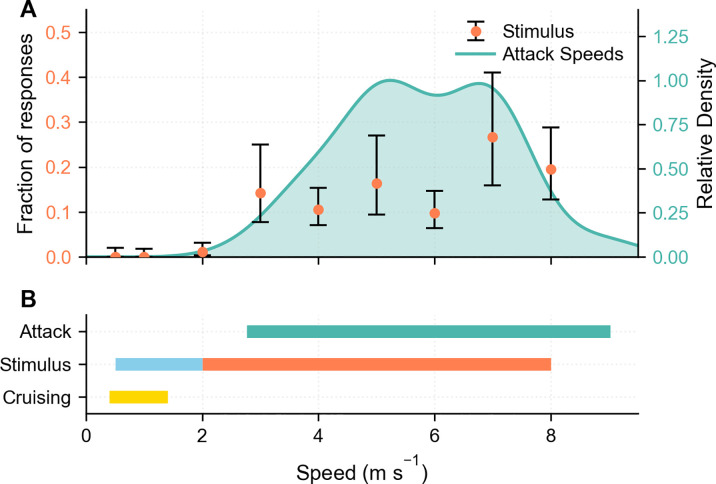
Responses per stimulus speed and comparison with natural swimming speeds of predators. A: The fraction of stimulus presentations at each speed that resulted in an escape response (orange points ±95% CI). Overlaid is a kernel density estimate of the 90th-percentile attack speeds from 61 recorded Bar Jack attacks (teal), representing the distribution of natural predator attack speeds. B: Speed ranges of natural attack speeds (teal) overlap with stimulus speeds that triggered escape responses (orange). Stimulus speed at lower speed ranges (light blue) did not trigger any responses, and overlapped with natural cruising speeds of predators (yellow).

Across all analyses, stimulus speed (LSp) emerged as the strongest predictor of escape responses. In the linear univariable model, higher stimulus speeds significantly increased response probability ($\beta =1.25\pm 0.15,p=7.0\times 10^{-18}$; S2 Table), and the effect remained highly significant in the multivariable model ($\beta =1.37\pm 0.17,p=3.2\times 10^{-16}$; S3 Table). Among all univariable models, it explained the largest proportion of variance ($R_{marginal}^{2}=0.19$). Response probability increased non-linearly with stimulus speed, showing a sharp rise above approximately $\approx 2.0\;m\;s^{-1}$ (Fig 2A), which was supported by model comparisons indicating that non-linear forms provided a better fit than the linear form (S4 Table).

This threshold-like pattern indicates that fish were largely unresponsive to slow-moving stimuli but responded reliably once the stimulus exceeded a certain speed. Such non-linear sensitivity is consistent with the activation properties of the Mauthner-cell escape circuit, which is tuned to rapid visual expansion [20,22,23]. This tuning may allow fish to avoid unnecessary escapes in non-threatening situations while maintaining high responsiveness to fast, potentially dangerous movements.

#### Speeds that trigger responses align with natural predator attack speeds.

Escape responses were only observed when stimulus speed exceeded $2.0\;m\;s^{-1}$, with the majority occurring at speeds of $3.0\;m\;s^{-1}$ or higher (Fig 2A). This behavioral threshold closely aligns with the attack speeds of Bar Jacks, which ranged from $2.7-9.0\;m\;s^{-1}$ (90th percentile; Fig 2B). Routine cruising speeds for *C. ruber* have not been reported, but the routine swimming speed of another carangid (e.g., Green Jack (*Caranx caballus*)) indicates routine speeds on the order of $\approx 1\;m\;s^{-1}$ or less for similarly sized fishes. These values are consistent with broader comparative studies in reef fishes, showing that attack movements are several times faster than routine swimming [24].

The close correspondence between response thresholds and natural predator attack speeds indicates that fish selectively respond to the speed of attacks rather than to ordinary cruising movements. This supports the idea that animals use the speed of approaching objects as a key cue for threat discrimination [14,15,29]. Avoiding responses to slower stimuli may help reduce false alarms in ambiguous situations, as slower movements are more likely to represent non-threatening situations. Our looming stimulus was a simplified, two-dimensional expanding disk rather than a realistic predator, which isolates the effect of approach speed but does not capture other cues a real predator provides. The weak response to slow stimuli is nonetheless consistent with threat-sensitive predator avoidance, in which prey grade their escape response to the level of threat and respond weakly to slow-moving or non-attacking predators [44,45]. Damselfish of the genus *Stegastes* show exactly this graded, threat-sensitive avoidance [46].

#### Spatial positioning influences visual access to the stimulus.

Variables that were specific to the experimental setup (specifically the distance to the stimulus (DSt) and the angle at which fish viewed the iPad (VA)) had a significant role in determining whether a fish initiated an escape response. Fish closer to the screen and viewing it from a smaller angle had a higher probability of responding.

These effects likely reflect the perceptual geometry of the setup. Because the looming stimulus was presented on a flat screen, it was most clearly visible when viewed from close range and at a smaller angle. At greater distances or wider angles, the screen became more reflective and the stimulus less distinct, making the threat harder to detect. This pattern is consistent with laboratory findings showing that response probability declines under reduced visual contrast and suboptimal viewing geometry [21,39].

For a detailed description of the effects, see S6 Appendix (Effect of experimental variables).

### Species show different behavioral strategies linked to spatial positioning

Species identity had a significant influence on the likelihood of escape responses. In the univariable model, Bicolor Damselfish had a substantially lower response probability than Brown Chromis (β=−1.66±0.37,p<0.001; S2 Table). This effect remained significant in the multivariable model (β=−1.10±0.48,p=0.02; S3 Table).

**Fig 3:**
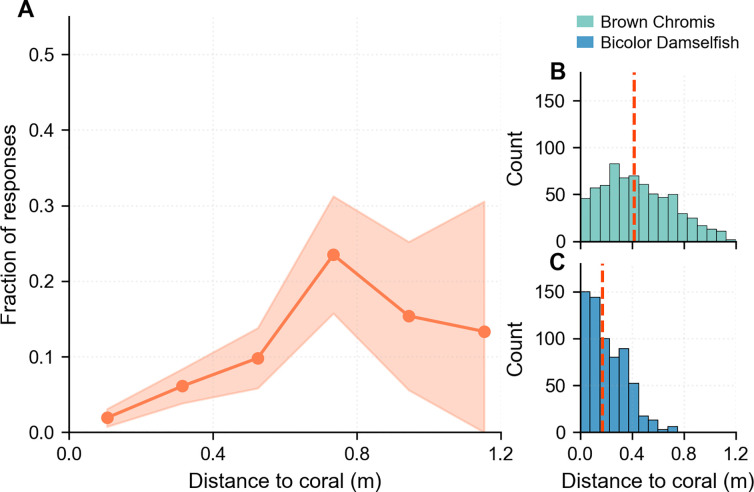
Species differences in escape responses are linked to distance from coral refuge. A: Fraction of fish responding across distance-to-coral bins, with shaded areas showing 95% CIs. Response probability increased with distance to the coral shelter, peaking at intermediate distances. B: Distribution of distances to coral for Brown Chromis. Red dashed line indicates median distance. C: Distribution of distances to coral for Bicolor Damselfish. Red dashed line indicates median distance.

However, the two species differed in their distance to the coral refuge. Brown Chromis tended to be farther from the coral (Fig 3B), while Bicolor Damselfish were typically located closer to shelter (Fig 3C). This difference was significant (Wilcoxon rank-sum test, *W* = 351,766, *p* < 0.001), confirming the correlation between species identity and distance to the coral. Distance to coral (DCo) itself had a significant positive effect on response probability in the univariable analysis (β=0.64±0.12,p<0.001; S2 Table), indicating that fish farther from shelter were more likely to flee (Fig 3A).

When both predictors were included in the same linear multivariable analysis, the effect of distance to the coral lost its statistical significance (β=0.11±0.17,p=0.54; S3 Table), although a log-transformed version improved model fit (ΔBIC=−4.92; S4 Table). In the final non-linear version, the effect remained non-significant (β=0.29±0.22,p=0.172; S5 Table). Including the log-transformed form in a species-only model substantially reduced the species coefficient (from β=−1.66 to β=−1.03) and improved model fit (ΔAIC=−18.4), but the species effect remained statistically significant (*p* = 0.01; S6 Table).

These results suggest that the two species differ in how they balance foraging against predation risk. Brown Chromis are zooplanktivores that forage higher in the water column, where plankton is more abundant but the coral refuge is farther away, a trade-off characteristic of planktivorous reef fishes [47]. They appear to compensate for this greater exposure with a higher tendency to flee, whereas Bicolor Damselfish remain close to the coral and rely less on escape. Notably, the species difference persists after accounting for distance to coral, indicating it is not driven by exposure alone but also reflects intrinsic differences, consistent with the territoriality and strong shelter-association of Bicolor Damselfish [27]. The two species may thus manage predation risk by different means: higher responsiveness in the more exposed species, and proximity to shelter in the more sedentary one.

### Social context had no significant effect on response

Contrary to expectations, the proximity of neighboring fish did not have a significant influence on the escape probability. In the univariable analysis (S2 Table), no effect was detected ($\beta =-0.65\pm 0.65,p=0.32,R_{marginal}^{2}=0.064$). In the multivariable model (S3 Table), the effect remained non-significant (β=−0.62±0.74,p=0.40). None of the tested interaction terms of neighbor proximity (involving distance to coral, species, and stimulus speed) improved the model fit (all adjusted p≥0.324, S7 Table).

We also tested two alternative measures for social context (the total angular area of neighbors in the focal fish’s visual field (NAS) and in the visual hemisphere containing the stimulus (${NAS}_{stim}$). Neither measure showed a significant effect in the multivariable analysis (see S7 Appendix), supporting the conclusion that social context did not affect escape responses.

These findings are surprising, as higher neighbor proximity has been shown to suppress responses in other studies, attributed to the reduced per-capita risk through dilution effects [28–30,48]. One possible explanation is that there was not enough variation in local density in our data to detect such an effect: the aggregations we observed were loosely grouped, and these species can form substantially denser aggregations than we sampled.

### Other variables did not affect the response

The other variables tested in this analysis had no significant effect on the response probability. The exact orientation of the fish relative to the stimulus (OA) did not have a significant effect on the response probability (S4 FigA), which is consistent with the fact that fish have a visual field of almost $360^{\circ }$ [49].

The order in which the stimulus was presented (Trial event; TEv) showed statistically significant spline terms in the final non-linear model (S5 Table), but the overall improvement in model fit compared to a linear form was negligible (ΔBIC=−1.34; S4 Table). In the linear multivariable model, the trial event number was not significant (*p* > 0.05; S3 Table), and the trend in response probability across successive trials did not show a consistent decline (S4 FigB). Together, these results suggest that habituation did not occur within the time frame of our study, consistent with the idea that habituation is limited under natural field conditions where threats are unpredictable and variable [37]. The statistically significant spline terms likely reflect minor fluctuations across the stimulus showings rather than a systematic change in responsiveness.

Finally, none of the prespecified two-way interactions (S7 Table) significantly improved model fit after controlling for the false discovery rate (Benjamini-Hochberg). The interaction between stimulus speed (LSp) and distance to the coral (DCo) lost its significance after the correction ($p_{adjusted}=0.165$). Although this interaction did not survive correction, its direction was suggestive: fish farther from the coral tended to respond at slightly lower stimulus speeds, which would fit with their greater exposure. All other interactions were non-significant in all analyses (p≥0.324).

## Conclusion

This study demonstrates that escape decisions in reef fish are primarily driven by visual information about the threat itself. Responses only occurred when stimulus speeds matched those of natural predator attacks, showing that these fish distinguish threatening from non-threatening motion on the basis of approach speed. This selectivity is consistent with the speed tuning of looming-sensitive escape circuits described in laboratory studies. The close correspondence indicates that effective threat discrimination can emerge from relatively simple perceptual processing rather than complex cognitive evaluation.

The effect of stimulus speed was consistent across species, but the two species differed in behavior. Bicolor Damselfish, which are territorial and shelter-dependent, stayed closer to the coral and were less likely to flee. Brown Chromis, which are more mobile and forage higher in the water column, were more exposed but compensated with a higher tendency to respond.

Surprisingly, the social context had no measurable effect on escape decisions, contrasting with previous research findings. This may reflect the loose aggregations we observed, which spanned too little variation in local density to reveal a dilution effect.

These findings highlight that escape behavior in reef fish is finely tuned to distinguish true danger from non-threatening motion, with species-specific behavior further shaping how individuals respond. This behavioral tuning to predator attack speeds is consistent with the looming-detection mechanisms characterized in other fishes, and shows how prey can achieve reliable threat discrimination in a complex, natural environment.

## Supporting information

S1 Fig Variation between trial sessions. A: Number of fish per site repetition. B: Number of fish per species across trial sessions. C: Number of datapoints per trial session. D: Neighbor proximity across trial sessions.
(TIF)

S2 FigEffect sizes and significance in linear models.Effect size estimates (β) and 95% CIs from (A) univariable models and (B) the multivariable model. Significant predictors are shown in dark blue, non-significant in gray.(TIF)

S3 Fig Escape response probabilities decline with distance to the stimulus and viewing angle. (A) Fraction of responses across distance to stimulus bins. (B) Fraction of responses across viewing angle bins. Shaded areas represent 95% CIs.
(TIF)

S4 Fig Effects of orientation angle and trial event number. (A) Fraction of responses across orientation angle bins. (B) Fraction of responses across trial event number. Although spline terms were statistically detectable, the effect was weak and inconsistent.
(TIF)

S1 Appendix Looming stimulus. Geometry of the looming stimulus and the conversion of simulated predator approach speed into on-screen expansion rate.
(PDF)

S2 Appendix Coral reconstruction. Procedure for reconstructing the three-dimensional coral geometry from annotated point clouds.
(PDF)

S3 Appendix Classification of individuals. Full description of how individual fish were classified into first responders, secondary responders, and non-responders based on their exposure to the stimulus, and the composition of the final dataset.
(PDF)

S4 Appendix Correlation between variables. Assessment of multicollinearity among continuous predictors using pairwise Pearson correlation coefficients.
(PDF)

S5 Appendix Justification of random effect. Model comparison justifying the inclusion of trial session ID as a random effect.
(PDF)

S6 Appendix Effect of experimental variables. Detailed results for the effects of distance to the stimulus and viewing angle on escape probability.
(PDF)

S7 Appendix Alternatives for neighbor proximity. Results for two alternative measures of social context (angular area of neighbors in the visual field and in the visual hemisphere containing the stimulus).
(PDF)

S1 Table Description of predictor variables used in the models. List of all experimental and ecological predictors, their abbreviations, and units.
(PDF)

S2 Table Results of univariable GLMMs predicting escape probability. Each model included a single predictor and a random intercept for trial session. Estimates are shown with standard errors, p-values, and marginal R^2^ values.
(PDF)

S3 Table Results of multivariable GLMM predicting escape probability. The model includes all predictors simultaneously, with random intercepts for trial session. Estimates, standard errors, z-values, and p-values are reported.
(PDF)

S4 Table Comparison of non-linear transformations for predictors in the context of the multivariable model. Comparison of BIC values for linear, log-transformed, and natural spline forms (2 or 3 *df*) of each predictor in the multivariable model. Best-fitting transformation per variable is indicated.
(PDF)

S5 Table Final model results using natural spline terms for stimulus speed and trial order. Results from the best-fitting model, including spline terms for stimulus speed and trial event, and log-transformations of distance to coral and distance to stimulus. Estimates, standard errors, z-values, and p-values are provided.
(PDF)

S6 Table Effect of adding individual predictors to the species-only model. Comparison of AIC values and species coefficients when adding each predictor separately to a baseline species-only model. Reduction in the species coefficient indicates predictors that account for species differences.
(PDF)

S7 Table Ecologically relevant interaction effects, added to the multivariable model with LSp as natural splines. Ecologically relevant two-way interactions were tested with likelihood ratio tests.
(PDF)

S8 Table Correlation matrix of continuous predictors using Pearson correlation coefficients. Pearson correlation coefficients among continuous predictors. All correlations are < 0.35, indicating no strong collinearity.
(PDF)
