# Supplementary material for: Reef fish escape responses selectively match predator attack speeds
Source: PLoS One. 2026 Aug 12;21(8):e0346465. doi: 10.1371/journal.pone.0346465 (PMC13465847; doi:10.1371/journal.pone.0346465)
Supplement: Supplementary file 1 [file pone.0346465.s005.pdf]

S1 Appendix. Looming stimulus

We generated looming stimuli to simulate a predator approach using a black disk that expanded on a white background. When viewed from a distance of 1 meter in front of the iPad screen (d_{screen}), the looming image corresponded to a predator with a radius of 4 cm ($r_{\text{predator}} = 0.04\,\text{m}$). Based on typical depth–length relationships for reef fishes [1], this radius corresponds to a predator approximately 30 cm in total length, which reflects a typical predator size for the 6–10 cm Brown Chromis and Bicolor Damselfish in our study. We simulated attacks with varying predator speeds ($v = 0.5$–$8.0\,\text{m}\,\text{s}^{-1}$) from a starting distance ($D_0 = 10\,\text{m}$). At the initiation of each simulated attack, the radius of the looming disk (R_{loom}, meters) at time t was calculated as:

$$R_{\text{loom}}(t) = \frac{d_{\text{screen}} \cdot r_{\text{predator}}}{D_0 - vt} \tag{1}$$

where t is time (s). To generate the image of the black disk, the calculated radius was converted to pixels using the pixel density of the iPad ($10{,}394\,\text{px}\,\text{m}^{-1}$). Videos of the looming stimuli were generated and played back at 120 frames per second.

References

1. Kulbicki M, Parravicini V, Mouillot D. Patterns and processes in reef fish body size. In: Mora C, editor. *Ecology of fishes on coral reefs*. Cambridge: Cambridge University Press; 2015. p. 104–115. Available from: https://doi.org/10.1017/CBO9781316105412.013.
