# Supplementary material for: Reef fish escape responses selectively match predator attack speeds
Source: PLoS One. 2026 Aug 12;21(8):e0346465. doi: 10.1371/journal.pone.0346465 (PMC13465847; doi:10.1371/journal.pone.0346465)
Supplement: Supplementary file 2 [file pone.0346465.s006.pdf]

S2 Appendix. Coral reconstruction

Annotation of the coral used the same software as for the fish (CVAT.ai Corporation, [1]). For every trial, multiple points on the coral were annotated and converted to 3D coordinates. These points were processed with a Delaunay-based triangulation, which connected neighboring points into a mesh of triangular faces; duplicate faces were then removed to obtain a clean surface. The resulting triangulated surface captured the geometry of each coral patch and provided a compact representation for the spatial analyses.

References

1. CVAT.ai Corporation. Computer vision annotation tool (CVAT). Version 2023.x; 2023. Available from: https://www.cvat.ai.
