# Supplementary material for: Reef fish escape responses selectively match predator attack speeds
Source: PLoS One. 2026 Aug 12;21(8):e0346465. doi: 10.1371/journal.pone.0346465 (PMC13465847; doi:10.1371/journal.pone.0346465)
Supplement: Supplementary file 3 [file pone.0346465.s007.pdf]

S3 Appendix. Classification of individuals

Response videos Fish in videos with a response were categorized based on their exposure to the stimulus and the frame of their response. First responders (FR, n = 106 before filtering; n = 95 included in this study) included all fish that initiated a response and any secondary responders that responded within five frames (20.83 ms) after the first responder. This time frame accounts for the sensory-motor delay required for visual information processing [1] and was determined as the minimum interval between a first responder and a secondary responder that could not directly see the stimulus in a video.

Secondary responders were further categorized based on their position relative to the stimulus. Those who viewed the stimulus at an angle smaller than 55° were classified as secondary responders who saw the looming stimulus (SRL, n = 143). Secondary responders outside this 55° angle were classified as secondary responders that could not see the looming stimulus and were, therefore, responding only to social cues from their neighbors (SRNL, n = 141). This distinction reflects the iPad screen's reflectivity at large angles, so the stimulus is no longer visible.

Non-responders were divided into categories based on their visibility of the stimulus as well. Non-responders within the 55° angle were classified as non-responders who saw both the looming stimulus and the escape responses of their neighbors (NRL-R, n = 335). Those outside the 55° angle were classified as non-responders who only observed the escape responses of neighbors (NRNL-R, n = 444).

Non-response videos For videos without any escape responses, individuals within the 55° angle were classified as fish that saw the stimulus (NRL-NR, n = 1341 before filtering; n = 1255 included in this study). Individuals outside the 55° angle were classified as fish that had no access to information about the stimulus (NRNL-NR, n = 1434). As no fish responded in the videos, neither type had neighbors responding.

Final dataset To ensure that the fish responded only to the stimulus, we compared the first responders in the response videos (FR, Fig 1E) with the unresponsive fish that saw the stimulus in the non-response videos (NRL-NR, Fig 1F). Both groups consisted of fish with non-responding neighbors, ensuring their response behavior was not influenced by the responses of others.

Four FR fish had viewing angles greater than 55°. Two had a viewing angle around 58°, which may reflect minor annotation errors. The other two had angles around 64°, but were positioned very close to the iPad, making the stimulus visible as it increased in size, since the viewing angle was measured from the center of the stimulus. All individuals were excluded from the final analysis.

The species group 'Other' consisted of a variety of species and families with small sample sizes: Basslets (including groupers and soapfish, *Serranidae*; n = 45), Wrasses (*Labridae*; n = 16), Parrotfish (*Scaridae*; n = 5), Snappers (*Lutjanidae*; n = 6), Doctorfish (*Acanthuridae*; n = 6), Dusky Damselfish (*Stegastes adustus*; n = 5), Sand tilefish (*Malacanthus plumieri*; n = 2), Flounders (*Bothidae*; n = 2), Angelfish (*Centropyge*; n = 2), Banded Butterflyfish (*Chaetodon striatus*; n = 1), Goatfish (*Mullidae*; n = 1), Boxfish (*Ostraciidae*; n = 1), and Yellowtail Hamlets (*Hypoplectrus chlorurus*; n = 1). Because of these small sample sizes, this group was excluded from the analysis.

The remaining dataset consisted of two species: Brown Chromis (*Chromis multilineata*; n = 696; FR = 83, NR = 613) and Bicolor Damselfish (*Stegastes partitus*; n = 654; FR = 12, NR = 642), which resulted in 95 FR fish and 1255 NRL-NR (referred to as NR) fish.

References

1. Martin BT, Gil MA, Fahimipour AK, Hein AM. Informational constraints on predator–prey interactions. *Oikos*. 2022;2022(10):e08143. Available from: https://doi.org/10.1111/oik.08143.
