# Supplementary material for: Reef fish escape responses selectively match predator attack speeds
Source: PLoS One. 2026 Aug 12;21(8):e0346465. doi: 10.1371/journal.pone.0346465 (PMC13465847; doi:10.1371/journal.pone.0346465)
Supplement: Supplementary file 4 [file pone.0346465.s008.pdf]

S4 Appendix. Correlation between variables

Multicollinearity among continuous predictors was assessed using pairwise Pearson correlation coefficients (see S8 Table). All pairwise correlations were low, with none exceeding 0.35, indicating the absence of strong linear relationships between variables. Based on this, all continuous predictors were retained for further analysis.
