# Supplementary material for: Reef fish escape responses selectively match predator attack speeds
Source: PLoS One. 2026 Aug 12;21(8):e0346465. doi: 10.1371/journal.pone.0346465 (PMC13465847; doi:10.1371/journal.pone.0346465)
Supplement: Supplementary file 5 [file pone.0346465.s009.pdf]

S5 Appendix. Justification of random effect

To verify whether the random effect significantly improved model fit, we compared a mixed-effects model with a corresponding fixed-effects model. Including the random intercept for trial session ID substantially improved model performance ($\Delta\text{BIC} = 92.16$). The likelihood ratio test confirmed that this effect was highly significant ($\chi^2 = 99.37, \text{df} = 1, p < 0.001$).
