# Supplementary material for: Reef fish escape responses selectively match predator attack speeds
Source: PLoS One. 2026 Aug 12;21(8):e0346465. doi: 10.1371/journal.pone.0346465 (PMC13465847; doi:10.1371/journal.pone.0346465)
Supplement: Supplementary file 6 [file pone.0346465.s010.pdf]

S6 Appendix. Effect of experimental variables

Spatial positioning relative to the stimulus (DSt and VA) played a significant role in determining whether a fish initiated an escape response. In the univariable analysis, distance to the stimulus (DSt) had a strong negative effect on response probability ($\beta = -1.06 \pm 0.15, p < 0.001$), explaining 15% of the variance ($R^2_{\text{marginal}} = 0.15$; S2 Table). This effect remained significant in the multivariable model ($\beta = -0.98 \pm 0.19, p < 0.001$; S3 Table), and a log-transformation of DSt substantially improved model fit ($\Delta\text{BIC} = -7.14$; S4 Table). The binned data show a decline in the fraction of responses with increasing distance to the stimulus (S3 Fig, panel A). The angle at which the stimulus was viewed also influenced response probability. Viewing angle (VA) had a negative effect in both the univariable ($\beta = -0.66 \pm 0.11, p < 0.001$; S2 Table) and multivariable models ($\beta = -0.64 \pm 0.14, p < 0.001$; S3 Table). This trend is also present in the binned visualization, where the fraction of responses declines with increasing viewing angle (S3 Fig, panel B).
