# Supplementary material for: Reef fish escape responses selectively match predator attack speeds
Source: PLoS One. 2026 Aug 12;21(8):e0346465. doi: 10.1371/journal.pone.0346465 (PMC13465847; doi:10.1371/journal.pone.0346465)
Supplement: Supplementary file 7 [file pone.0346465.s011.pdf]

S7 Appendix. Alternatives for neighbor proximity

As an alternative to neighbor proximity (NPro), two other variables representing social context were tested: the total angular area of the neighboring fish in the focal fish's eye (NAS), and the total angular area in the side of the visual field containing the stimulus (NAS_{stim}). Both were calculated from the size of the neighboring fish and their distance to the focal fish. Because the fish were small, their measured sizes were prone to annotation error, so neighbor proximity (which does not depend on fish size) was the more reliable measure and was used in the primary analysis.

When the two variables were tested in univariable analysis, NAS_{stim} had a significant influence on the response probability ($\beta = -0.47 \pm 0.15, p < 0.05$). In the multivariable analysis, this effect became non-significant. This loss of significance likely reflects the confounding effect of a strong predictor in the model, specifically the distance to the stimulus, with which NAS_{stim} has a weak correlation ($r = 0.22$). If fish were further from the stimulus, they had more neighbors in the visual hemisphere containing the stimulus. In both analyses, NAS was non-significant.
