# Supplementary material for: Reef fish escape responses selectively match predator attack speeds
Source: PLoS One. 2026 Aug 12;21(8):e0346465. doi: 10.1371/journal.pone.0346465 (PMC13465847; doi:10.1371/journal.pone.0346465)
Supplement: Supplementary file 8 [file pone.0346465.s012.pdf]

S1 Table. Description of predictor variables used in the models. List of all experimental and ecological predictors, their abbreviations, and units.

**Variable**	**Symbol**	**Description**	**Units**
Species	Sp	Species (*Bicolor Damselfish* vs. *Brown Chromis*)	Categorical
Orientation angle	OA	Orientation of fish relative to the stimulus	° (0–180)
Viewing angle	VA	Angle at which the fish views the stimulus	° (0–180)
Distance to coral	DCo	Distance to the closest point on the coral	m
Distance to stimulus	DSt	Distance to the center of the stimulus	m
Stimulus speed	LSp	Looming speed of the stimulus	$\mathrm{m\,s^{-1}}$
Trial event	TEv	Repetition of stimulus event in trial	Ordinal
Neighbor proximity	NPro	Summed squared reciprocal distance to all neighbors	$\mathrm{m^{-2}}$
