# Supplementary material for: Reef fish escape responses selectively match predator attack speeds
Source: PLoS One. 2026 Aug 12;21(8):e0346465. doi: 10.1371/journal.pone.0346465 (PMC13465847; doi:10.1371/journal.pone.0346465)
Supplement: Supplementary file 9 [file pone.0346465.s013.pdf]

S2 Table. Results of univariable GLMMs predicting escape probability. Each model included a single predictor and a random intercept for trial session. Estimates are shown with standard errors, p-values, and marginal R^2 values.

**Variable**	**Estimate**	**Std. Error**	**p-value**	$\mathbf{R}^2_{\text{marginal}}$
Species (Sp)	–1.6570	0.3674	6.49×10^{-6}	0.1108
Orientation angle (OA)	–0.0596	0.1207	0.622	0.0006
Viewing angle (VA)	–0.6639	0.1053	2.83×10^{-10}	0.0663
Distance to coral (DCo)	0.6386	0.1178	5.85×10^{-8}	0.0634
Distance to stimulus (DSt)	–1.0630	0.1449	2.22×10^{-13}	0.1523
Stimulus speed (LSp)	1.2550	0.1457	7.03×10^{-18}	0.1861
Trial event (TEv)	–0.1810	0.1195	0.130	0.0053
Neighbor proximity (NPro)	–0.6510	0.6515	0.318	0.0640
