# Supplementary material for: Reef fish escape responses selectively match predator attack speeds
Source: PLoS One. 2026 Aug 12;21(8):e0346465. doi: 10.1371/journal.pone.0346465 (PMC13465847; doi:10.1371/journal.pone.0346465)
Supplement: Supplementary file 10 [file pone.0346465.s014.pdf]

S3 Table. Results of multivariable GLMM predicting escape probability. The model includes all predictors simultaneously, with random intercepts for trial session. Estimates, standard errors, z-values, and p-values are reported.

**Variable**	**Estimate**	**Std. Error**	**z-value**	**p-value**
Intercept	–3.6891	0.6443	–5.726	1.03×10^{-8}
Species (Sp)	–1.0994	0.4812	–2.285	0.022
Orientation angle (OA)	–0.0455	0.1446	–0.315	0.753
Viewing angle (VA)	–0.6385	0.1379	–4.629	3.68×10^{-6}
Distance to coral (DCo)	0.1059	0.1723	0.615	0.539
Distance to stimulus (DSt)	–0.9836	0.1904	–5.165	2.40×10^{-7}
Stimulus speed (LSp)	1.3717	0.1680	8.164	3.24×10^{-16}
Trial event (TEv)	–0.2589	0.1576	–1.643	0.100
Neighbor proximity (NPro)	–0.6228	0.7369	–0.845	0.398
