# Supplementary material for: Reef fish escape responses selectively match predator attack speeds
Source: PLoS One. 2026 Aug 12;21(8):e0346465. doi: 10.1371/journal.pone.0346465 (PMC13465847; doi:10.1371/journal.pone.0346465)
Supplement: Supplementary file 11 [file pone.0346465.s015.pdf]

S4 Table. Comparison of non-linear transformations for predictors in the context of the multivariable model. Comparison of BIC values for linear, log-transformed, and natural spline forms (2 or 3 *df*) of each predictor in the multivariable model. Best-fitting transformation per variable is indicated.

**Variable**	**BIC (linear)**	**BIC (log)**	**BIC (spline 2 *df*)**	**BIC (spline 3 *df*)**	**Best form**
Orientation angle (OA)	402.665	402.685	407.798	414.945	Linear
Viewing angle (VA)	402.665	406.729	406.898	413.451	Linear
Distance to coral (DCo)	407.587	402.665	408.084	411.766	Log
Distance to stimulus (DSt)	409.803	402.665	411.602	412.182	Log
Stimulus speed (LSp)	428.864	407.657	407.042	402.665	Spline (3 df)
Trial event (TEv)	402.665	405.192	404.945	401.328	Spline (3 df)
Neighbor proximity (NPro)	402.665	404.191	409.695	416.149	Linear
