# Supplementary material for: Reef fish escape responses selectively match predator attack speeds
Source: PLoS One. 2026 Aug 12;21(8):e0346465. doi: 10.1371/journal.pone.0346465 (PMC13465847; doi:10.1371/journal.pone.0346465)
Supplement: Supplementary file 12 [file pone.0346465.s016.pdf]

S5 Table. Final model results using natural spline terms for stimulus speed and trial order. Results from the best-fitting model, including spline terms for stimulus speed and trial event, and log-transformations of distance to coral and distance to stimulus. Estimates, standard errors, z-values, and p-values are provided.

**Variable**	**Estimate**	**Std. Error**	**z-value**	**p-value**
Intercept	–21.22	4.84	–4.38	1.16×10^{-5}
Species (Sp)	–0.87	0.54	–1.62	0.105
Orientation angle (OA)	–0.08	0.16	–0.51	0.608
Viewing angle (VA)	–0.72	0.15	–4.69	2.78×10^{-6}
Log(Distance to coral) (DCo)	0.29	0.22	1.36	0.172
Log(Distance to stimulus) (DSt)	–1.08	0.19	–5.55	2.79×10^{-8}
Stimulus speed (LSp; spline 1)	11.26	2.54	4.43	9.31×10^{-6}
Stimulus speed (LSp; spline 2)	37.67	9.47	3.98	6.90×10^{-5}
Stimulus speed (LSp; spline 3)	9.59	2.04	4.71	2.45×10^{-6}
Trial event (TEv; spline 1)	1.86	0.76	2.46	0.014
Trial event (TEv; spline 2)	–3.84	1.31	–2.93	0.0034
Trial event (TEv; spline 3)	–3.43	0.88	–3.91	9.18×10^{-5}
Neighbor proximity (NPro)	–1.06	0.83	–1.28	0.200
