# Supplementary material for: Reef fish escape responses selectively match predator attack speeds
Source: PLoS One. 2026 Aug 12;21(8):e0346465. doi: 10.1371/journal.pone.0346465 (PMC13465847; doi:10.1371/journal.pone.0346465)
Supplement: Supplementary file 13 [file pone.0346465.s017.pdf]

S6 Table. Effect of adding individual predictors to the species-only model. Comparison of AIC values and species coefficients when adding each predictor separately to a baseline species-only model. Reduction in the species coefficient indicates predictors that account for species differences.

**Predictor added**	**AIC**	**Species effect**	**SE**	**p-value**
None (baseline)	546.8	–1.66	0.367	6.5×10^{-6}
Orientation angle (OA)	548.6	–1.66	0.368	6.5×10^{-6}
Viewing angle (VA)	513.3	–1.59	0.386	3.7×10^{-5}
Distance to coral (DCo; log)	528.4	–1.03	0.409	0.01
Distance to stimulus (DSt; log)	489.5	–1.41	0.391	3.3×10^{-4}
Stimulus speed (LSp; splines)	429.7	–1.71	0.410	3.0×10^{-5}
Trial event (TEv; splines)	544.0	–1.66	0.370	7.7×10^{-6}
Neighbor proximity (NPro)	547.2	–1.65	0.367	6.6×10^{-6}
