# Supplementary material for: Reef fish escape responses selectively match predator attack speeds
Source: PLoS One. 2026 Aug 12;21(8):e0346465. doi: 10.1371/journal.pone.0346465 (PMC13465847; doi:10.1371/journal.pone.0346465)
Supplement: Supplementary file 14 [file pone.0346465.s018.pdf]

S7 Table. Ecologically relevant interaction effects, added to the multivariable model with LSp as natural splines. Ecologically relevant two-way interactions were tested with likelihood ratio tests.

**Variable 1**	**Variable 2**	**Effect Var 1**	**Effect Var 2**	χ^2	**Raw p-value**	**Adjusted p-value**
LSp	DCo	Splines (3 *df*)	Log	9.13	0.0276	0.165
LSp	Sp	Splines (3 *df*)	Categorical	5.02	0.170	0.324
LSp	NPro	Splines (3 *df*)	Linear	4.78	0.189	0.324
NPro	Sp	Linear	Categorical	1.53	0.216	0.324
DCo	Sp	Log	Categorical	1.00	0.318	0.382
NPro	DCo	Linear	Log	0.56	0.454	0.454
