# Supplementary material for: Reef fish escape responses selectively match predator attack speeds
Source: PLoS One. 2026 Aug 12;21(8):e0346465. doi: 10.1371/journal.pone.0346465 (PMC13465847; doi:10.1371/journal.pone.0346465)
Supplement: Supplementary file 15 [file pone.0346465.s019.pdf]

S8 Table. Correlation matrix of continuous predictors using Pearson correlation coefficients. Pearson correlation coefficients among continuous predictors. All correlations are < 0.35, indicating no strong collinearity.

**Variable**	**OA**	**VA**	**DCo**	**DSt**	**LSp**	**TEv**	**NPro**
OA		0.043	–0.020	0.014	0.065	0.018	0.036
VA			–0.293	0.305	0.036	0.023	0.021
DCo				–0.277	0.071	–0.004	–0.073
DSt					–0.015	0.030	0.006
LSp						–0.026	0.023
TEv							0.028
NPro							
